# Three Ingredients of Safflower Alleviate Acute Lung Injury and Inhibit NET Release Induced by Lipopolysaccharide

**DOI:** 10.1155/2020/2720369

**Published:** 2020-02-29

**Authors:** Yun-peng Wang, Yu Guo, Ping-shan Wen, Zhen-zhen Zhao, Jian Xie, Kai Yang, Qing Yang, Jia-feng Wang, Xiao-ming Deng

**Affiliations:** Faculty of Anesthesiology, Changhai Hospital, Naval Medical University, 168 Changhai Road, Shanghai 200433, China

## Abstract

Xuebijing injection is a Chinese herb compound to treat sepsis in China, but it contains many different kinds of components, and each component may have different effects in treating sepsis. The present study was performed to investigate the effect of three ingredients of Xuebijing, safflor yellow A (SYA), hydroxysafflor yellow A (HSYA), and anhydrosafflor yellow B (AHSYB), in lipopolysaccharide- (LPS-) induced acute lung injury (ALI). LPS (10 mg/kg) was injected intratracheally to induce acute lung injury in mice, which were then treated with SYA, HSYA, and AHSYB. The blood, bronchoalveolar lavage fluid (BALF), and lung tissues were collected to detect degree of lung injury, level of inflammation, and neutrophil extracellular traps (NETs). In vitro experiments were performed using HL-60 cells stimulated with phorbol myristate acetate (PMA). Lung injury induced by LPS was alleviated by SYA, HSYA, and AHSYB as demonstrated by the histopathologic test. The three components inhibit LPS-induced elevation of the levels of inflammatory factors and wet-to-dry weight ratio as well as the amount of protein and cells in the BALF. They also induced a remarkably less overlay of myeloperoxidase (MPO) and histone in the immunofluorescence assay and reduced level of MPO-DNA complex in plasma. The in vitro assay showed a similar trend that the three components inhibited PMA-induced NET release in neutrophil-like HL-60 cells. Western blot demonstrated that phosphorylation of c-rapidly accelerated fibrosarcoma (c-Raf), mitogen-activated protein kinase ERK kinase (MEK), and extracellular signal-regulated kinase (ERK) in the lungs of LPS-challenged mice, and PMA-treated HL-60 cells were all significantly reduced by SYA, HSYA, and AHSYB. Therefore, our data demonstrated that three components of XBJ, including SYA, HSYA, and AHSYB, showed a protective effect against LPS-induced lung injury and NET release.

## 1. Introduction

Sepsis is defined as life-threatening organ dysfunction caused by a dysregulated host response to infection [1]. Acute lung injury (ALI)/acute respiratory distress syndrome (ARDS) is present in a large proportion of septic patients and may lead to an increased mortality [2]. Neutrophils play an important role in innate immunity. When the host is challenged by the pathogenic microorganisms, neutrophils will be mobilized and migrate to the infectious locus driven by the concentration gradient of chemokines such as interleukin-8 (IL-8), C5a, fMLP, and leukotriene B4 [3]. The neutrophils recruited to the infected sites can kill the pathogens by phagocytosis and then be eliminated by macrophages [4]. On the other hand, it is believed that the activation, infiltration, and delayed elimination of neutrophils have a major effect on the progression of ALI/ARDS [5]. Neutrophil extracellular traps (NETs) are primarily composed of cellular-free DNA, histones, and globular proteins such as myeloperoxidase (MPO) [6]. They work as a valuable antimicrobial defense mechanism; however, accumulating evidences demonstrate that excessive formation of NETs contributes to the pathogenesis of several diseases, such as appendicitis, acute lung injury, systemic lupus erythematosus, and sepsis [7–9]. During these conditions, excessive histones and MPO are toxic to epithelial cells and endothelial cells [9], and NETs may be involved in the pathogenesis of ALI/ARDS as a potential therapeutic target against lung injury.

Xuebijing (XBJ) is a traditional Chinese herbal prescription which is widely used in treating sepsis in China [10]. It has been also reported to have a positive effect on the treatment of ALI/ARDS related to sepsis [11, 12] and community-acquired pneumonia [13]. XBJ contains the following five main Chinese herbs, including safflower, red peony root, Szechuan Lovage Rhizome, Radix Salviae Miltiorrhizae, and Chinese angelica. Among these herbs, safflower is commonly used for improving microcirculation, relieving pain, and inhibiting inflammation in traditional medicine in China, Middle East, and other countries [14]. We speculated that the effects of safflower on microcirculation and inflammation might be useful in relieving sepsis-induced pulmonary inflammation, but we did not know which molecules within safflower provided these effects. Some researchers analyze the main ingredients of XBJ by mass spectrometry and identified several different ingredients from XBJ, among which safflor yellow A (SYA), hydroxysafflor yellow A (HSYA), and anhydrosafflor yellow B (AHSYB) were derived from safflower [15]. Therefore, the present study was performed to investigate the effect of SYA, HSYA, and AHSYB on lipopolysaccharide- (LPS-) induced lung injury in mice.

## 2. Materials and Methods

### 2.1. Experimental Animals and Cells

Male C57BL/6J mice that were 6-8 weeks of age and free of specific pathogens were obtained from the Research Animal Center of Navy Medical University (Shanghai, China). All experimental animals used in this study were approved by the Institutional Animal Care and Use Committee of the Navy Medical University. All experiments were performed in accordance with the relevant guidelines and regulations.

The cell line used was the human leukemia cell line, HL-60. The cells were cultured in RPMI-1640 medium (Invitrogen, Carlsbad, CA, USA) supplemented with 10% heat-inactivated fetal bovine serum (FBS) (Invitrogen, Carlsbad, CA, USA) and maintained at 37°C with 5% CO_2_. With this line, differentiation to mature granulocytes can be induced by compounds such as dimethyl sulfoxide (DMSO) (Sigma, St. Louis, MO, USA).

### 2.2. In Vivo Experiments

The acute lung injury model was established as described previously [16]. Briefly, the mice were anesthetized with sevoflurane (Hengrui, Lianyungang, Jiangsu, China). After exposing the trachea, a trimmed sterile 31-gauge needle was inserted into the tracheal lumen. LPS (Sigma, St Louis, MO, USA) diluted in endotoxin-free saline was intratracheally (IT) injected at a dose of 10 mg/kg in 50 *μ*l saline.

To treat ALI mice, SYA (ChromaDex, Irvine, CA, USA), HSYA (Santa Cruz, Dallas, TX, USA) and AHSYB (Nature Standard, Shanghai, China) was dissolved in saline and intraperitoneally administered immediately after the injection of LPS. All these three compounds were given at 5 × 10^−5^ mol/kg in 400 *μ*l saline [17]. The materials required for the experiment were obtained after 24 hours.

### 2.3. In Vitro Experiments

The HL-60 cells cultured in 10% FBS medium were treated with DMSO (1.25%) to induce the differentiation into neutrophil-like cells [18]. After five days, the cells were stimulated with PMA (MedChemExpress, Monmouth Junction, NJ, USA) (100 ng/ml, dissolved in DMSO) or medium or DMSO. Then, SYA, HSYA, and AHSYB (160 mg/l) were added into the medium [19]. Three hours later, cells were harvested after centrifugation while the supernatant was collected for other use.

### 2.4. Histology and Immunofluorescence

Lungs were fixed in 4% paraformaldehyde solution for more than 48 hours. Subsequently, the lungs were embedded in paraffin and sectioned. Sections were then stained with hematoxylin and eosin (HE) after deparaffinization. Alveolar congestion, hemorrhage, aggregation of inflammatory cells, and the thickness of the alveolar walls were assessed under a light microscope. The result was semiquantified by two independent pathologists according to the criteria reported previously [20, 21].

For immunofluorescence assay, sections were incubated overnight with various primary antibodies at 4°C after blocking. And secondary antibodies with different fluorescence colors were added to the sections for 1 h at room temperature. After being rinsed and mounted with glycerol, the sections were recorded using fluorescence microscopy.

### 2.5. Enzyme-Linked Immunosorbent Assays (ELISAs) of Cytokine

Mouse blood was collected from the postocular venous plexus and centrifuged. The plasma was harvested to detect the cytokine level using TNF-*α* or IL-6 ELISA kits (Invitrogen, Carlsbad, CA, USA) following the manufacturer's instructions. The results were measured using a microtiter plate reader at 450 nm.

### 2.6. Lung Wet-to-Dry Weight Ratio

The wet weight of the lungs was measured instantly after lung tissues were harvested. Then, the lungs were incubated in an oven at 80°C for 3 days to remove moisture, and the dry weight was measured. The wet-to-dry weight (W/D) ratio was calculated to assess the edema.

### 2.7. BALF Analysis

The BALF was obtained by intratracheal injection with 1 ml cold PBS. Then, the BALF was centrifuged at 1500 rpm for 10 min. The protein concentration in the supernatant was assessed with a BCA detection kit (Thermo Scientific, Rockford, IL, USA). Briefly, solution A and solution B were mixed and then added into each sample. After incubation in 37°C for 30 min, the OD values at the length of 540 nm were read. Then, the protein concentration of each sample was obtained according to the standard curve.

The cells in the BALF were collected and stained with anti-Ly6G-PE and anti-CD11b-APC (eBiosciences, San Diego, CA, USA) to detect neutrophils by flow cytometry. Flow cytometry data was obtained using a FACSCanto II flow cytometer (BD Bioscience, San Jose, CA, USA) and analyzed by FlowJo software, version 7.6.1 (Tree Star, Ashland, OR, USA).

### 2.8. Detection of MPO-DNA

To quantify NETs in mouse plasma and in cell culture supernatant, a capture ELISA based on MPO associated with DNA was applied [22]. For the capture antibody, 5 *μ*g/ml anti-MPO Ab (Invitrogen, Carlsbad, CA, USA) was coated onto 96-well plates (dilution 1 : 500 in 50 *μ*l) overnight at 4°C. After washing 3 times (300 *μ*l each), 20 *μ*l of samples was added to the wells with 80 *μ*l incubation buffer containing a peroxidase-labeled anti-DNA antibody (Cell Death ELISA PLUS, Roche, Indianapolis, IN, USA; dilution 1 : 25). The plate was incubated for 2 hours, shaken at 300 rpm at room temperature. After 3 washes (300 *μ*l each), 100 *μ*l peroxidase substrate (ABTS) was added. Absorbance at 405 nm wavelength was measured after 20 minutes of incubation at room temperature in the dark. Values for soluble NET formation were expressed as fold increase in absorbance above control.

### 2.9. Western Blot Analysis

The lung tissues or the HL-60 cells were lysed in RIPA Buffer (1 mM EDTA pH 8.0, 50 mM Tris-HCl pH 8.0, 2% SDS, and 5 mM DTT), and their protein concentration was determined by the BCA assay (Beyotime, Shanghai, China). The total protein was separated by a SDS-PAGE gel and transferred to PVDF (polyvinylidene fluoride) membranes (Immobilon, Merck KGaA, Darmstadt, Germany) and then blocked with 5% nonfat dry milk in PBST (phosphate-buffered saline with Tween) with a pH of 7.5. The membranes were incubated with primary antibodies: c-Raf, P-c-Raf, MEK, P-MEK, ERK, and P-ERK (Cell Signal Technology, Beverly, MA, USA, diluted at 1 : 1000) for 4 hours at room temperature or overnight at 4°C. The secondary antibodies, goat anti-mouse IgG-HRP and goat anti-rabbit IgG-HRP (Engibody Biotechnology, Milwaukee, WI, USA, diluted at 1 : 2000), were incubated for 2 hours at room temperature. Finally, the protein bands were detected by an enhanced chemiluminescence kit (Pierce, Rockford, IL, USA). The relative semiquantitative analysis was based on optical density with ImageJ software, version 1.51 (Rawak Software, Inc. Germany).

### 2.10. Statistical Analysis

Statistical analysis was conducted with GraphPad Prism 7 (GraphPad Software Inc., CA, USA) using one-way analysis of variance (ANOVA) and the 2-tailed Student *t*-test. All data were presented as mean values ± standard error of mean. *P* values < 0.05 were considered statistically significant.

## 3. Results

### 3.1. SYA, HSYA, and AHSYB Alleviate LPS-Induced Lung Histopathologic Changes

**Figure 1:**
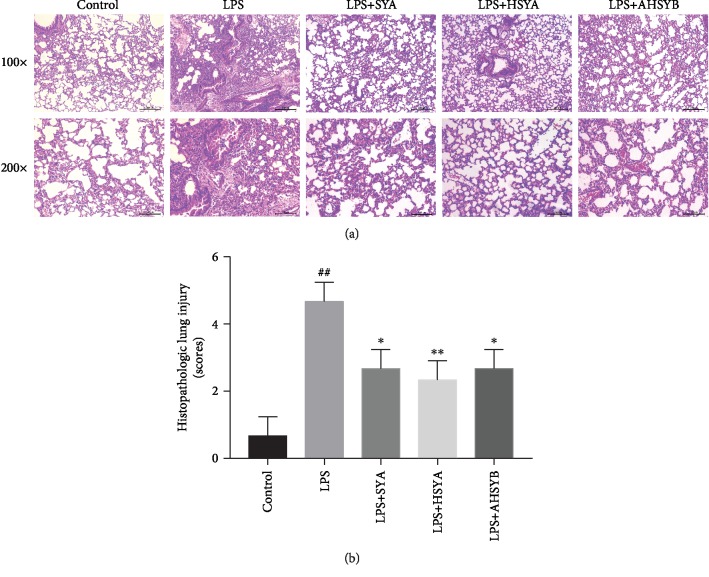
Effects of SYA, HSYA, and AHSYB on LPS-induced histopathologic changes in the lungs. (a) 24 hours after LPS injection, the lungs in each group were prepared for histological evaluation. Representative histological section of the lungs was stained by HE staining, magnification (100x, bar = 200*μ*m; 200x, bar = 100*μ*m). (b) The lung injury scores were determined. The values presented are mean ± SEM, ^##^*P* < 0.01 vs the control group; ^#^*P* < 0.05 vs the control group; ^∗^*P* < 0.05 vs the LPS group; ^∗∗^*P* < 0.01 vs the LPS group.

Lung injury was assessed by HE staining of lung tissue 24 hours after LPS injection. As shown in Figure 1(a), in the LPS group, the lung tissue of the mice was severely damaged with the destroyed alveolar structure, edematous and thickened pulmonary septum, and a large number of inflammatory cells infiltrated in the interstitium and alveoli. Although inflammatory reactions were also observed in the lung tissues of the SYA group, HSYA group and AHSYB group, the impairment was significantly lighter, with less inflammatory cells infiltrated and milder lung interstitial damage (Figure 1(a)). The pathological scores indicated that SYA, HSYA, and AHSYB could alleviate the lung injury induced by LPS (Figure 1(b)).

### 3.2. SYA, HSYA, and AHSYB Suppress LPS-Induced Inflammatory Responses in the Lungs

**Figure 2:**
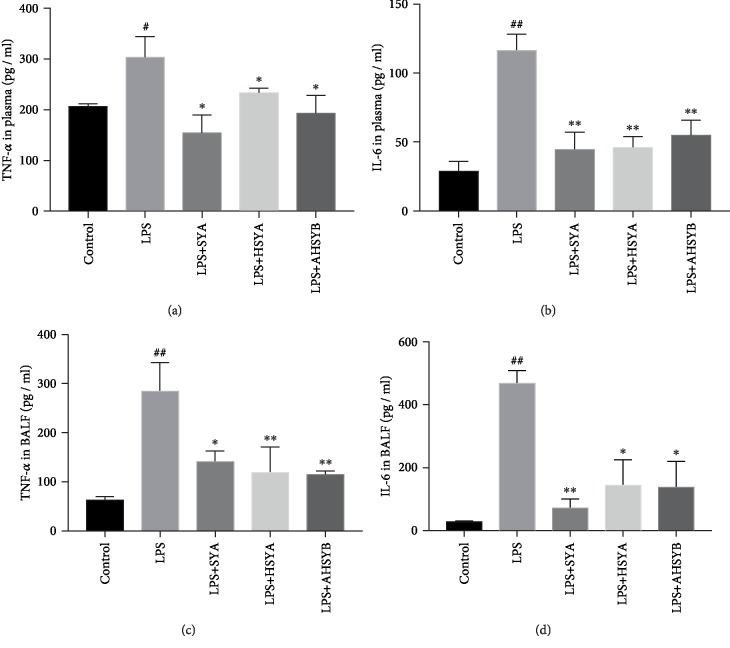
Effects of SYA, HSYA, and AHSYB on the production of inflammatory cytokines in LPS-induced ALI. (a) TNF-*α* in plasma 24 h after ALI. (b) IL-6 in plasma 24 h after ALI. (c) TNF-*α* in BALF 24 h after ALI. (d) IF-6 in BALF 24 h after ALI. The values presented are mean ± SEM, ^##^*P* < 0.01 vs the control group; ^#^*P* < 0.05 vs the control group; ^∗^*P* < 0.05 vs the LPS group; ^∗∗^*P* < 0.01 vs the LPS group.

Inflammatory cytokines play a key role in LPS-induced ALI. We measured the level of TNF-*α* and IL-6 in plasma and BALF to indicate the severity of inflammatory responses in the lungs. Compared with the control group, LPS significantly increased the secretion of TNF-*α* and IL-6 both in plasma and BALF, and this trend was reversed by SYA, HSYA, and AHSYB (Figure 2). Thus, we can infer that SYA, HSYA, and AHSYB can suppress LPS-induced lung inflammatory cytokine production.

### 3.3. SYA, HSYA, and AHSYB Protect the Permeability of the Lungs in LPS-Induced ALI

**Figure 3:**
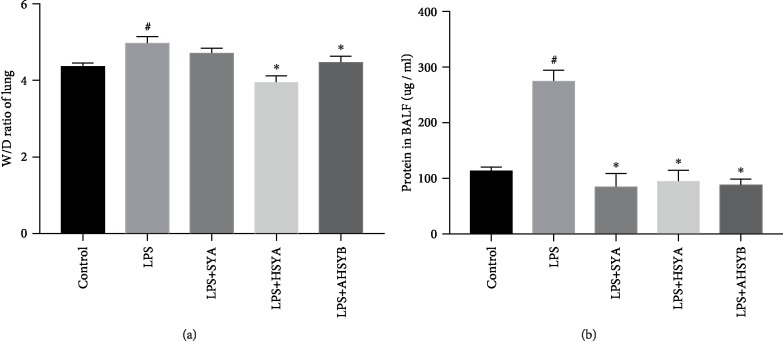
Effects of SYA, HSYA, and AHSYB on the edema in LPS-induced ALI. (a) Lung tissues were weighed to calculate the W/D ratio. (b) BCA assay was used to determine the total protein concentration in BALF. The values presented are mean ± SEM, ^#^*P* < 0.05 vs the control group; ^∗^*P* < 0.05 vs the LPS group.

In ALI, the permeation of macromolecules and fluid into the interstitium was increased when the endothelial cell barrier was damaged. The wet-to-dry weight ratio of the lungs and total protein concentration of BALF are a signal to assess the edema in ALI. 24 hours after LPS injection, the wet-to-dry weight ratio of the lungs was increased, and HSYA and AHSYB blocked the increase while SYA did not show this effect (Figure 3(a)). But SYA, HSYA, and AHSYB all significantly reduced the concentrations of total proteins in BALF after LPS injection (Figure 3(b)). These data implied that SYA, HSYA, and AHSYB relieved the severity of increased lung permeability in ALI caused by LPS.

### 3.4. SYA, HSYA, and AHSYB Block the Inflammatory Cell Infiltration in the Lungs

**Figure 4:**
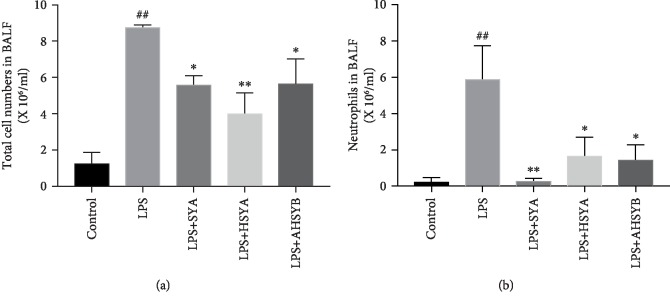
Effects of SYA, HSYA, and AHSYB on inflammatory cells induced by LPS. (a) The total cells detected by flow cytometry in BALF. (b) The neutrophils detected by flow cytometry in BALF. The values presented are mean ± SEM, ^##^*P* < 0.01 vs the control group; ^#^*P* < 0.05 vs the control group; ^∗^*P* < 0.05 vs the LPS group; ^∗∗^*P* < 0.01 vs the LPS group.

In the progression of ALI, neutrophils accumulate in the lungs following the chemokine release from macrophages and mediate lung injury. Thus, the effect of SYA, HSYA, and AHSYB on the infiltration of inflammatory cells in the lungs was investigated. It was observed that LPS increased the number of total cells and neutrophils in BALF 24 hours after LPS injection. However, after the administration of SYA, HSYA, and AHSYB, the number of total cells and neutrophils in BALF was reduced markedly (Figure 4). These data indicated that SYA, HSYA, and AHSYB could inhibit the inflammatory cell infiltration in ALI caused by LPS.

### 3.5. SYA, HSYA, and AHSYB Inhibit NET Formation in Impaired Lungs

**Figure 5:**
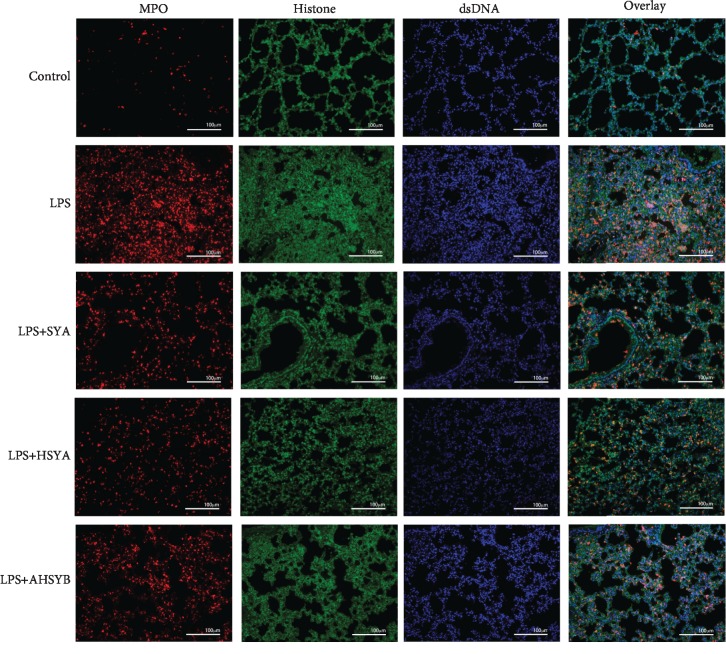
Effects of SYA, HSYA, and AHSYB on LPS-induced lung NET production in the lungs (MPO: red; histone: green; dsDNA: blue; bar = 100*μ*m).

NETs mainly consist of DNA, histone, and MPO; thus, we stained the lung section with these primary antibodies. The LPS group mouse lung section contained more histone and MPO compared with the control group, while SYA, HSYA, and AHSYB reversed this tendency (Figure 5). These results reflect directly that SYA, HSYA, and AHSYB could reduce the production of NETs.

### 3.6. SYA, HSYA, and AHSYB Decreased NETs In Vivo and In Vitro

**Figure 6:**
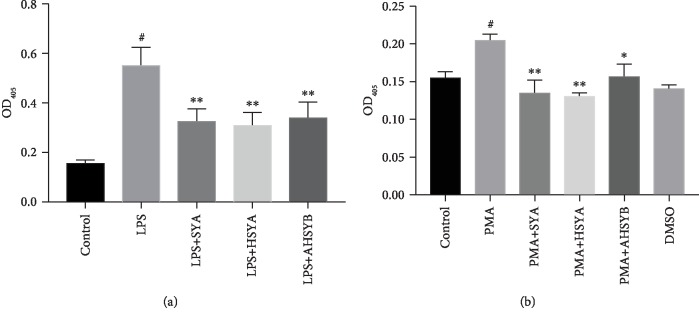
Effects of SYA, HSYA, and AHSYB on NETs. (a) Effects of SYA, HSYA, and AHSYB on plasma MPO-DNA complex in LPS-induced ALI mice. The values presented are mean ± SEM, ^#^*P* < 0.05 vs the control group; ^∗∗^*P* < 0.01 vs the LPS group. (b) Effects of SYA, HSYA, and AHSYB on MPO-DNA complex in PMA-induced HL-60 cells. ^#^*P* < 0.05 vs the control group; ^∗^*P* < 0.05 vs the PMA group; ^∗∗^*P* < 0.01 vs the PMA group.

Generally, NETs can be detected by ELISA with a capture antibody against MPO-DNA. In vivo, we found that absorbance at 405 nm wavelength was increased in the LPS group compared with the control group. Treatment with SYA, HSYA, and AHSYB eliminated these alterations. The similar results were also observed in PMA-stimulated HL-60 cells in vitro. SYA, HSYA, and AHSYB decreased the upregulation of OD_405_ induced by addition of PMA to the medium (Figure 6).

### 3.7. SYA, HSYA, and AHSYB Inhibit the Raf/MEK/ERK Signaling Pathway

**Figure 7:**
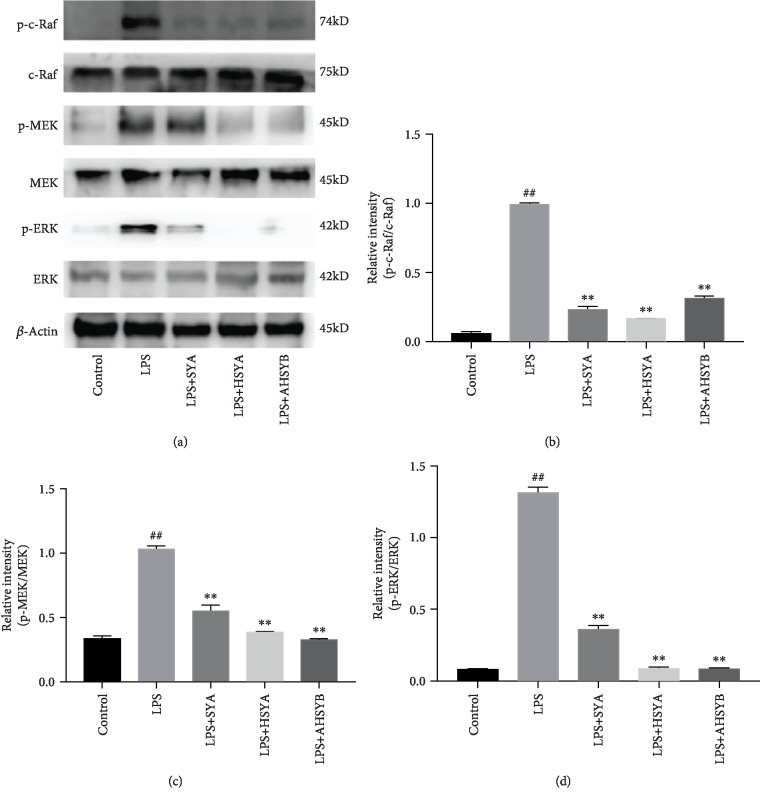
Effects of SYA, HSYA, and AHSYB on Raf/MEK/ERK signaling pathway expression in the lungs. (a) Protein levels of p-c-Raf, c-Raf, p-MEK, MEK, p-ERK, and ERK in lung homogenates were evaluated by western blot analysis 24 h after LPS injection. (b–d) Densitometric analyses of the relevant bands. The values presented are mean ± SEM. ^##^*P* < 0.01 vs the control group; ^#^*P* < 0.05 vs the control group; ^∗^*P* < 0.05 vs the LPS group; ^∗∗^*P* < 0.01 vs the LPS group.

The activation of the Raf/MEK/ERK signaling pathway is necessary for NET formation, and thus, we examined the effect of SYA, HSYA, and AHSYB on phosphorylation level of c-Raf, MEK, and ERK in lung homogenate and in HL-60 cell lysate to provide further evidence of the change in pathways of NET formation. As shown in Figure 7, the levels of the phosphorylated c-Raf, MEK, and ERK were markedly increased after LPS challenge. SYA, HSYA, and AHSYB inhibited the activation of these signaling molecules.

**Figure 8:**
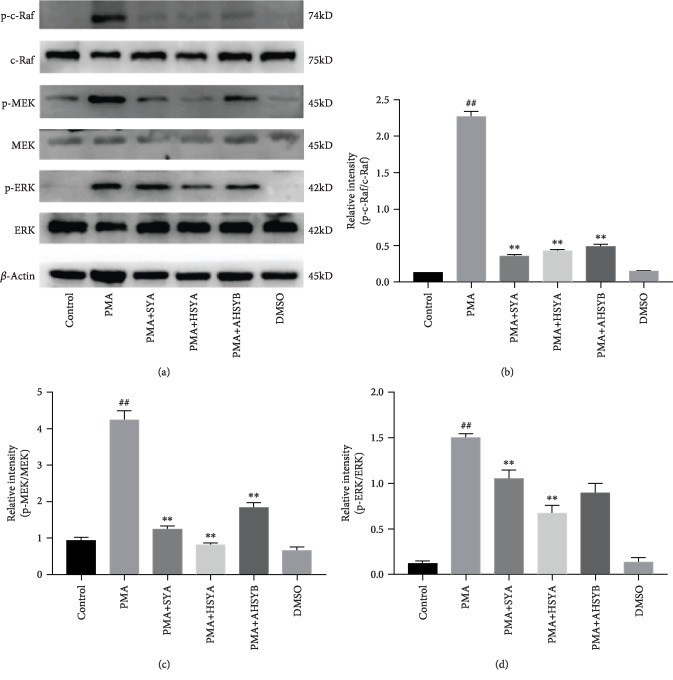
Effects of SYA, HSYA, and AHSYB on Raf/MEK/ERK signaling pathway expression in cells. (a) Protein levels of p-c-Raf, c-Raf, p-MEK, MEK, p-ERK, and ERK in cell homogenates were assessed by western blot analysis 3 h after PMA stimulation. (b–d) Densitometric analysis of the relevant bands was performed. The values presented are mean ± SEM. ^##^*P* < 0.01 vs the control group; ^#^*P* < 0.05 vs the control group; ^∗^*P* < 0.05 vs the LPS group; ^∗∗^*P* < 0.01 vs the LPS group.

Similar trends of the phosphorylated level of c-Raf, MEK, and ERK were observed in the cell experiment. As shown in Figure 8, SYA, HSYA, and AHSYB inhibit the Raf/MEK/ERK signaling pathway which is essential for NET formation.

## 4. Discussion

Our present study demonstrated that three main constituents of safflower identified in XBJ, including SYA, HSYA, and AHSYB, could alleviate pulmonary inflammation and impairment induced by LPS. They can also reduce the formation of NETs and inhibit the Raf/MEK/ERK pathway in lungs challenged by LPS and in neutrophil-like HL-60 cells stimulated by PMA.

SYA, HSYA, and AHSYB are among the main constituents of safflower, which is one of the five main herbs in XBJ. Safflower is a traditional herb in Chinese medicine with antioxidant and anti-inflammatory effects. It has also been reported to protect against ischemic heart disease [23]. Chinese medicine is always composed of several different kinds of herbs, and each herb may have many biological molecules with various therapeutic effects. It is important for us to understand the exact effect of each biological molecule within these herbs for developing monomeric drug. Therefore, the present study was performed to investigate the role of SYA, HSYA, and AHSYB on LPS-induced lung injury and the underlying pathophysiological changes.

It is really interesting to find that the three constituents of safflower can inhibit NET formation. The process of NET release represents a special way of neutrophil cell death called “NETosis” [24]. The main protein components of NETs are histones, followed by granzymes and peptides, including neutrophil elastase (NE), MPO, cathepsin G, leukocyte protease 3 (PR3), lactoferrin, lysozyme C, and calprotectin [25]. In subsequent studies, NETs were found to be associated with a variety of diseases, including infectious [26, 27] and non-infectious diseases [28–30]. NETs protect the host by capturing and killing pathogens; however, excessive NET formation can be harmful [31]. It has been reported that NETs play an important role in intravascular thrombosis, disseminated intravascular coagulation, and multiple organ dysfunction, and these pathological processes can increase the incidence and mortality of sepsis [32, 33]. Thus, NETs are a promising target against ALI/ARDS, and the three constituents of safflower may provide a new insight for drug development.

The three constituents of safflower have been investigated in other animal models. It was confirmed that SYA could protect neonatal rat cardiomyocytes against anoxia/reoxygenation injury in vitro [34]. There have been a large number of studies showing the protective effects of HSYA on the cardiovascular system [35–37], nervous system [38, 39], liver [40, 41], lung [42, 43], and others. It was also found to inhibit certain tumors [44, 45] and has a certain impact on metabolism [46]. But there are currently few studies on the pharmacological effect of AHSYB. Our present study is the first to investigate the three constituents on LPS-induced lung injury simultaneously since they are the main components of XBJ.

XBJ is a Chinese patent medicine that was approved for treating sepsis. Its antiendotoxin and anti-inflammatory effects have been demonstrated in many animal experiments and clinical trials for the treatment of sepsis. A meta-analysis which consists of 16 RCTs suggested that XBJ may improve the 28-day mortality rate, APACHE scores, WBC count, and body temperature of septic patients without serious adverse events [10]. Furthermore, researchers have studied that XBJ can ameliorate ALI induced by sepsis via several different mechanisms. Wang et al. found that XBJ may inhibit the releases of proinflammatory cytokines through the HMGB1/RAGE axis [11]. Another study indicated that XBJ may have an impact on lung capillary leakage by upregulating Tollip expression and inhibiting lung inflammatory responses and oxidant stress and protecting lung permeability [12]. Other mechanisms may include the shift of macrophage from M1 to M2 phenotype [47], inhibition of IL-6 expression, and maintenance of IL-10 expression at the protein and mRNA levels [48]. Our present study may increase our understanding of the effects and mechanisms of XBJ during sepsis and organ injury.

It has been proved that XBJ injection had a significant efficacy in treating sepsis because of its bioactive components which can inhibit the NF-*κ*B pathway [49]. SYA and HSYA are two of these bioactive components. Yang et al. found that SYA exerts an anti-inflammatory effect on BV2 microglia, possibly through the TLR-4/p38/JNK/NF-*κ*B signaling pathway [50]. Besides, it has been reported that HSYA inhibits the phosphorylation of NF-*κ*B and p38 MAPK in BV2 cells in OGD/reoxygeneration (OGD/R) conditions [51]. In addition, HYSA has been reported to activate the HIF-1*α*/VEGF signaling pathway and then reduce the production of ROS and maintain the integrity of mitochondrial membrane [19]. Raf/MEK/ERK may be another target pathway of SYA, HSYA, and AHSYB to alleviate the inflammatory responses.

There are several limitations in our current study. First, we proposed that SYA, HSYA, and AHSYB might protect LPS-induced ALI via NETosis. However, there may be some other potential targets for SYA, HSYA, and AHSYB which are involved in this protective effect. Moreover, NETs can also be generated by a pathway independent of Raf/MEK/ERK [52, 53]. The mechanism underlying the effect of SYA, HSYA, and AHSYB on NET formation may be very complicated, and our data showed that Raf/MEK/ERK might be involved in this effect. Second, the drug dosage used in this study is based on the previous literature [50, 51, 54–56]. We have not investigated the dose-effect of these three agents. Further pharmacokinetic and toxicological studies may be required to validate the medicinal value of SYA, HSYA, and AHSYB.

## 5. Conclusion

In summary, this study found that SYA, HSYA, and AHSYB, the three main active components of safflower identified in Xuebijing, can alleviate lung injury induced by LPS and NET formation. Although we do not know the exact mechanism how these constituents act with the inflammatory responses in neutrophils, the inhibition of the Raf/MEK/ERK pathway and NET formation might be involved in the mechanism of the protective effect of SYA, HSYA, and AHYSB against LPS-induced lung injury.

## Data Availability

The data including HE staining of the lungs, expression of TNF-alpha and IL-6, wet-to-dry weight ratio of lungs, total protein concentration of BALF, number of total cells and neutrophils in BALF, immunofluorescence of NETs in the lungs, level of NETs, and expression of the Raf/MEK/ERK pathway used to support the findings of this study are included within the article.
